# MLPAstats: An R GUI package for the integrated analysis of copy number alterations using MLPA data

**DOI:** 10.1186/1471-2105-12-147

**Published:** 2011-05-11

**Authors:** Alejandro Cáceres, Lluís Armengol, Sergi Villatoro, Juan R González

**Affiliations:** 1 Center for Research in Environmental Epidemiology, Doctor Aiguader 88, Barcelona 08003, Spain; 2 Institut Municipal d'Investigació Mèdica, Doctor Aiguader 88, Barcelona 08003, Spain; 3 Quantitative Genomic Medicine Laboratories, Doctor Aiguader 88, Barcelona 08003, Spain; 4 Center for Genomic Regulation, Doctor Aiguader 88, Barcelona 08003, Spain; 5 CIBER Epidemiología y Salud Pública, Doctor Aiguader 88, Barcelona 08003, Spain

## Abstract

**Background:**

Multiplex-Dependent Probe Amplification (MLPA) is a cost-effective experimental method for candidate gene studies, aimed at the identification of copy number alterations. The analysis of such genetic variants, from electropherogram peak intensities, involves two main stages. First, peak normalization for each probe is required to remove the contribution of probe size to peak intensity. Second, the statistical significance of peak alteration between case and control samples is estimated. A number of methods have been proposed in each step with varying levels of complexity and precision. However, there is no single framework from which the results of each method and possible combinations at each step can be assessed.

**Results:**

We present MLPAstats, an R package designed to integrate the methods for exploring different analysis scenarios in a reliable way. A GUI has been developed to allow researchers to find their optimal analysis strategy.

**Conclusions:**

MLPAstats is an analysis tool that promotes the use of cost-effective MLPA suitable for candidate gene studies. Its R implementation allows future methods to be easily incorporated, while its GUI will facilitate its use by non-expert analysts. A vignette describing a set-by-step tutorial is also available with the package.

## Background

Recent research shows that copy number variations (CNV) are an important form of inter-individual genetic difference in the human population. Currently, a popular approach to identifying novel mechanisms of genetic predisposition to disease is to search for recurrent differences in the copy number of certain genes between control and affected populations (the case-control design).

CNV-based studies consist of variant detection, followed by testing for association with the phenotypes of interest. Association testing requires appropriate estimation of copy number status for each genetic probe and for each sample in the population. Here, we deal with the detection of CNVs and refer the reader to other sources for a discussion of association testing, which may take uncertainty in CNV calling in to account (CNVassoc package submitted for publication).

The detection of CNVs have been made possible by recent advancements in experimental technology and data analysis. In the context of genome-wide studies, a large amount of genetic regions are scanned with platforms such as aCGH, Illumina or Affymetrix. Complex segmentation algorithms are required for the identification of relevant genetic sequences. As a consequence, the benefit of testing large number of markers is limited by the power to accurately detect copy number events.

Studies with targeted genes can be performed with more precise experimental techniques, such as Multiplex Ligation-dependent Probe Amplification (MLPA), Quantitative Multiplex PCR of Short Fluorescent (QMPSF) or Multiplex Amplifiable Probe Hybridization (MAPH). Particularly, MLPA, a semi-qualitative technique, is able to determine gains or losses in the copy number of the targeted genomic regions [1]. Some of its most attractive features are its easy implementation and low cost. In a case-control design, the method can analyze up to 50 different genomic sequences.

The multiplex PCR amplification, on which MLPA is based, produces an electropherogram of peak intensities for each probe and subject. The hight of a given peak depends not only on the number of copies of the targeted region but also on its probe size. Therefore, the copy number alteration of the gene between case and control samples can be measured from the variation of the peak intensity, accounting for probe size. Early analyses of MLPA data did not considered within-subject variability of the probes. In the case of having replicates, this variability can be assessed and incorporated in the stages of the analysis as shown by Gonzalez et al. [2]. This decreases type I error while increasing statistical power.

Few methods have been developed to detect CNVs from MLPA data, some of which are offered in Coffalyser, an Excel package recommended by the manufacturer. Although researchers are encouraged to use Coffalyser for its usability, the software requires a Microsoft Office license to operate and, more importantly, it does not incorporate recent developments on the analysis of MLPA data, such as analysis with replicates [2]. In a previous study, Gerdes and colleagues [3] implemented a fixed work-flow for the interassay evaluation analysis of MLPA kits. More recently, van Eijk et al. [4] developed MLPAinter, a tool for the visualization and the quality control of MLPA data. The tool is particularly useful for dealing with high number of sample sizes and identifying stable reference probes. However, as a stand alone unit, it does not allow the incorporation of normalization and estimation of dosage ratios by third parties, and its current implementation includes a threshold method that does not perform statistical inferences on the CNV status of the probes.

In this article, we present MLPAstats, a free package written on R with a GUI that includes both common and more state-of-the art methodologies. MLPAstats features analyses with and without replicates, covering a wide range of data acquisitions and experimental designs. Given the variety of possible strategies, the software allows researchers to explore the optimal analysis for their data. This includes not only the methods but also the selection of reference probes and replicate samples. We first describe the implementation of the software. Then, we illustrate the package using samples with and without replicates http://www.creal.cat/jrgonzalez/software.htm. We use the GUI in the first case and the command line in the second sample. And finally, we compare MLPAstat and Coffalyser on a third data set.

## Implementation

The two main steps of an MLPA analysis are the normalization and the inference of copy number alterations. Normalization by probe size is a data pre-processing step that takes into account the systematic non-biological variation between samples. Variation can arise from the size and nature of the probe, differences in experimental conditions in each sample, and PCR efficiency. These factors need to be considered before an assessment of the differences in copy number between groups can be performed.

The two most common methods of normalization are based on average (sum) of peak intensities and regression models. An estimate of the normalization factor is computed and applied to the original data set. In the case of no replicates, individual normalization factors are commonly taken as the total sum of peak intensities for each subject. If replicates for the subject have been collected, then the normalization coefficients can be computed form the peak average across replicates.

Given that the peak intensity decreases with probe size, the normalization factor can be modeled as a function of it. A straightforward model is to consider a linear dependence between probe peaks and sizes. Typically, reference probes are chosen to guide the normalization.

Normalized peak intensities are then used to assess differences in copy number between case and control samples. A simple approach is to examine whether the ratio between case and control intensities falls outside predefine thresholds. Ratios lower than 0.7 are considered losses and ratios over 1.33 are gains in copy number [5,6].

A more suitable approach is Regression-Enhanced MLPA (REX-MLPA) where the regression between case and control probes is computed with given confidence intervals. Outliers of the confidence region are identified as case probes with altered copy number. Starting with a regression on reference probes, the method iteratively includes test probes that are within the confidence intervals, to re-estimate the regression. A final model is fitted with all the probes within the interval. The probes outside the confidence limits are considerer significantly altered in copy number between case and control samples.

If replicates are available, for each subject in the sample, then the within-subject variation of reference probes can be used in a mixed model that includes an overall mean and group effect. In such model, a probe with altered copy number in the case group has an estimate that falls outside the confidence interval. The interval is obtained from the mean of the differences between control samples, and the estimate of the error variance.

## Results

MLPAstats is written in R and can be freely downloaded from http://www.cran.r-project.org. For guidance on how to install and launch the software, see the user's manual (additional file 1), also a vignette distributed with the package. The manual demonstrates a reproducible step-by-step analysis of experimental data presented by [2].

MLPAstats features a GUI based application that is launched from the R command line:

> gui.mlpa()

**Figure 1:**
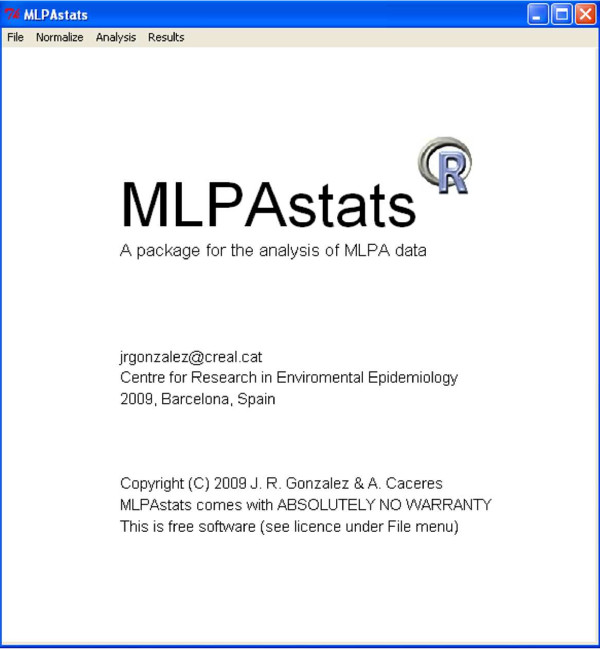
**MLPAstats main GUI window**.

Figure 1 shows MLPAstats main window. The user can interactively load and set up the analysis session. This process is carried out by creating an Ms.Rdata file in which data and analysis results are saved at any stage, to be recovered in future sessions. Various analyses strategies can be explored and saved, so the results are easily reproduced. Alternatively, the main functions of the software can be called from the R command line, when data has been conveniently loaded and set-up on the R global environment.

In the following sections, we first illustrate the use of the GUI with a data set that has no replicates, and then we show how to call the main functions from the command line, using a sample data set with replicates. Note, however, that both data sets can be analyzed either way.

### Normalization

The normalization procedures implemented in MLPAstats include different aspects of the relationship between peak intensity and probe size. In some occasions, a set of reference probes are sampled to guide the normalization. Normalization options are:

1. *sum peaks controls*: Only reference probes are used for the normalization. All peak intensities are divided by the sum of reference probe peaks. This procedure corrects a global factor between the control and case groups.

2. *sum peaks all*: If the profiles of *all *probe peaks across groups seem equivalent up to a factor, then normalization can be performed by dividing the peaks by the sum of all probes [1].

3. *slope correction*: Larger probe sizes have lower peak intensity. Removal of this effect is done with a linear regression between the size and the probe intensity. The regression is preformed on the reference probes.

4. *non linear: *If replicates are available, an estimation of the within-subject error can be taken into account. An exponential decay is further considered to remove the inverse relationship between probe size and peak intensity.

**Figure 2:**
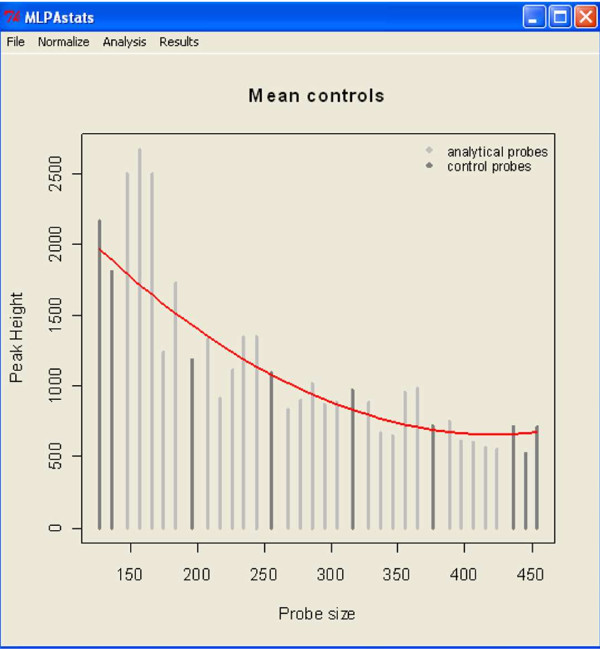
**Normalize reults for BRCA data**.

To demonstrate the normalization procedure on data without replicates, a *BRCA *sample data can be loaded from the *File *menu under *load demo*. The data is from a breast cancer study (P002 BRCA1) provided by NGRL-Manchester. It consists on a collection of 34 probes for 10 case and 5 control samples. Nine of the probes are used as reference probes for the normalization step. Loading the data will create the Ms.Rdata file on the present working directory and will update the current status of the data to be normalized. Since this data has no replicates it can only be normalized with *slope correction *or *sum peaks *options, found under the *Normalize *main menu. The result of the normalization is displayed in Figure 2 and obtained by selecting from the main window: *Results*→*plot*→*normalization*→*mean controls*.

The normalization updates the Ms.Rdata file and sets up the data for the assessment of differences in copy number alterations.

### Copy Number Alteration

Assessing statistical significance of the peak differences between case and control samples is the main objective of MLPAstats. From these inferences, probes are catalogued as having a relative gain (1), loss (-1) or no change (0) in their copy number status between samples.

Three analyses are implemented in MLPAstats:

1. *threshold: *A direct comparison between case and control samples is performed. Ratios between reference and test peaks that are found outside predefined thresholds are considered as altered. Default threshold values are 0.7 for deletions and 1.33 for gains. Different thresholds can be considered.

2. *REX-MLPA: *Regression enhanced MLPA is based on an iterative regression between case and control samples. Starting with control probes the regression defines the threshold for which target probes are considered to have no change in copy number. Such probes are used to re-estimate the thresholds. Test probes that are finally outside the confidence intervals are classified as having either a gain or a loss in copy number.

3. *mixel-model: *The error in the peak intensities for the control probes can be computed if subject replicates are available. The comprehensive model that takes into account between probe, probe-test (main effect), and random variability can substantially increase the precision of CNV calling [2].

**Figure 3:**
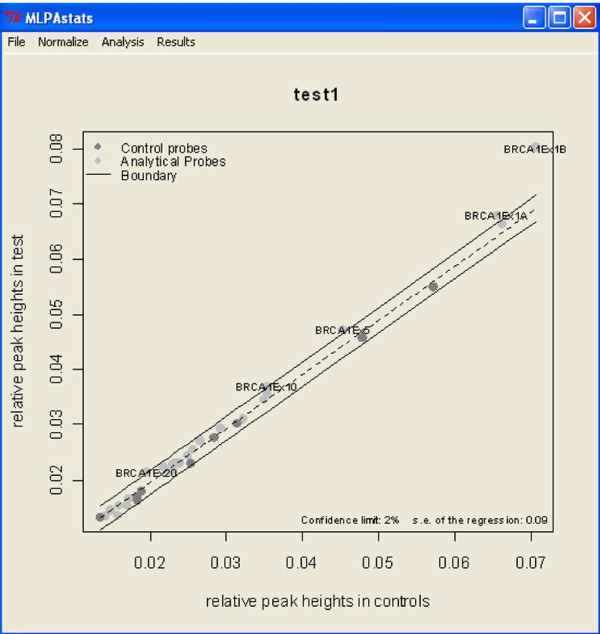
**Analysis Results using REX-MLPA**.

After normalization, MLPAstats computes the copy number status (-1,0,1) for each case probe relative to the control probes. For the previously normalized BRCA data, which has no replicates, only the *threshold *and the *REX-MLPA *method can be used. Under the *Analysis *menu the inference methods are listed and followed by a window where relevant parameters are specified. The results of a REX-MLPA are shown in Figure 3. The plot shows the scattered plot of normalized intensities for case against control samples.

### MLPAstats**from the command line**

Here we illustrate the call of the main functions, for normalization and copy number alteration inference, from the command line. We use a data set with replicates, MLPAvalidation, presented in [2]. The data sample is also available for a GUI analysis from *load demo *under the File menu.

In the command line the data is loaded and set up with the instructions

> data(MLPAvalidation)

> mlpa.dat<-setupMLPA(controls, cases, size, reference.probes)

The data in controls, cases, sizes and reference.probes, obtained from MLPAvalidation, has the required format for setupMLPA. Normalization is then performed by calling the function mlpaNorm

> mlpa.norm<-mlpaNorm(mlpa.dat, method="sums.peaks.controls")

mlpa.norm can be plotted or taken forward into the analysis. The function mlpa, with the appropriate analysis option (mixel-model) for data with replicates

>ans<-mlpa(mlpa.norm, method="mixed-model")

**Figure 4:**
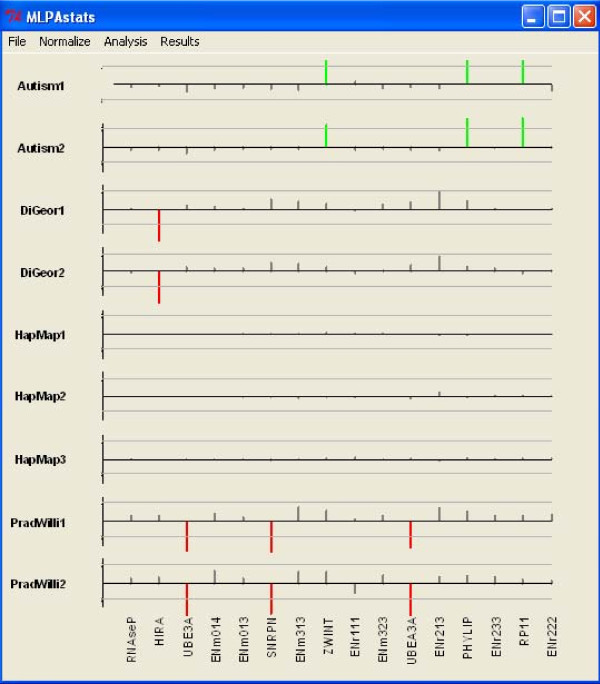
**Analysis Results with replicates using mixed-model option**. Markers are shown on the horizontal line and subjects on the vertical line. Markers with red (green) had a significant loss (gain) in copy number between case and test samples.

>ans

gives the copy number alterations for each probe. An example of a result display is shown in Figure 4. Equal results are obtained following the corresponding steps in the GUI.

### comparison between MLPAstats and Coffalyser

We performed a comparison between these two softwares using the sample data distributed with Coffalyserhttp://old.mlpa.com/coffalyser/. This is a 17 DMD samples (13 good sample runs and 2 control runs) and reference "MLPA mix P034 MLPA probemix lot 1105, 0505, 1004". We performed the analysis following the instructions manual. We filtered and analyzed with the option "Control Probe Analysis". Then, from the "info", "reference runs" and "sample runs" of the Excel sheets, we collected all the information needed to run MLPAstats. For that analysis, we normalized with "sum peaks control" and estimated dosage ratios with "REX-MLPA". We chose that analysis strategy so it would be similar to the one we used for Coffalyser. Table 1 shows the results of both analyses.

**Table 1:** Copy Number Alteration

D1.03.01831DMD		
	**MLPAstats**	**Coffalyser**

1357-L1005	1	1

1361-L1009	1	1

1363-L1011	-1	0

1365-L1013	1	1

1373-L1021	1	1

1385-L1033	1	1

1713-L1281	1	1

1715-L1283	1	1

1718-L1286	1	1

1897-L1008	-1	0

1954-L1574	1	1

**D1.04.04280DMD**		

	**MLPAstats**	**Coffalyser**

1385-L1033	-1	-1

1715-L1283	-1	-1

1718-L1286	-1	-1

**D1.05.05580DMD**		

	**MLPAstats**	**Coffalyser**

1355-L1615	1	0

1370-L1287	-1	-1

1374-L1288	-1	-1

1378-L1026	-1	-1

1382-L1030	-1	-1

1390-L1038	-1	Amb

1711-L1279	1	0

1717-L1285	-1	-1

Prediction of copy number alteration using MLPAstats and Coffalyser for the test sample DMD P034-1, distributed with Coffalyser. Key: 1-Gain, 0-Normal, -1-Loss, Amb-Ambiguos. Only samples with copy number alteration are shown.

We observed that MLPAstats found all the copy number variants reported by Coffalyser, and also detected two more variants in the first and third sample. Therefore, while both analyses are comparable to each other, we found that the MLPAstats implementation has an increased power. Although it should be noted that MLPAstats reports the ambiguous status on the third sample as a loss.

We believe that a main advantage of MLPAstats is, however, its ability to treat multiple replicas of the samples, for which Coffalyser has no options. In addition, a clear division between normalization and estimation of dosage rations allows for easy optimization of the analysis strategy. Finally, on the user's side, the GUI and its vignette are easier to follow, and the code is publicly open to incorporate future developments.

## Conclusions

Given the accessibility of MLPA experiments, it is desirable that its data analysis is also usable. The interface of MLPAstats has been designed to combine and compare different analyses that can be stored and retrieved at any stage. Effort has also been put into establishing an easy interaction with the user; in particular, into setting up the analysis and displaying results. The GUI should encourage the wide use of MLPAstats, regardless of previous knowledge in R.

The user's manual, distributed with the package, further illustrates how to set up and analyze experimental data.

## Availability and requirements

1. Project name: MLPAstats

2. Project home page: http://www.creal.cat/jrgonzalez/software.htm and http://www.cran.r-project.org

3. Operating system(s): Platform independent

4. Programming language: R

5. R Dependencies: nmle, boot, tcltk, tkrplot, pixmap

6. License: GPL or newer

## Authors' contributions

AC wrote the manuscript and implemented the GUI. LA and SV tested the software, reviewed the manuscript and designed the experiment with replicas. JRG designed the R package, wrote the R functions (including methods and classes), illustrated the use of the algorithms using the two real examples and reviewed the manuscript. All authors have read, and approved the final manuscript.

## Supplementary Material

Additional file 1 **Document that shows a step-by step manual of MLPAstats**. Click here for file
