# Supplementary material for: MLPAstats: An R GUI package for the integrated analysis of copy number alterations using MLPA data
Source: BMC Bioinformatics. 2011 May 11;12:147. doi: 10.1186/1471-2105-12-147 (PMC3118163; doi:10.1186/1471-2105-12-147)
Supplement: Additional file 1 — Document that shows a step-by step manual of MLPAstats. [file 1471-2105-12-147-S1.PDF]

MLPAstats User's Guide

Alejandro Cáceres and Juan R. González

February 11, 2011

Center for Research in Environmental Epidemiology (CREAL)
Institut Municipal d'Investigació Mèdica (IMIM)

`jrgonzalez@creal.cat`
`http://www.creal.cat/jrgonzalez/software.htm`

Contents

1 Installation **1**

2 Loading Demo **2**
2.1 Exploring the Ms object 2
2.2 Making you own Ms 4

3 Normalization **6**
3.1 Updating Ms 7

4 Analysis of Dosage Ratios **7**
4.1 Results on Ms 8

5 MLPAstats from the command line **11**

6 Acknowledgments **12**

Abstract

MLPAstats is software, written in R, design for the analysis of differences in CNVs using multiplex-dependent probe amplification (MLPA) data. It is freely available at http://www.cran.r−project.org and its methods described by Gonzalez el al. [1]. The software is provided with a graphical user interface (GUI), that facilitates its use, in particular, for those not familiar with R. Here we present a step-by-step analysis of the data studied in reference [1].

1 Installation

MLPA stats is written for R (http://www.r−project.org.) which can be downloaded from http://www.cran.r−project.org.. The package is installed using:

```
> install.packages("MLPAstats")
```

You can load the package on R by typing

```
> library(MLPAstats)
```

MLPAstats functions can be accessed by a GUI that is opened with the instruction

```
> gui.mlpa()
```

```
wellcome to MLPAstats
<Tcl>
```

This launches MLPAstats main window as shown in figure (1).

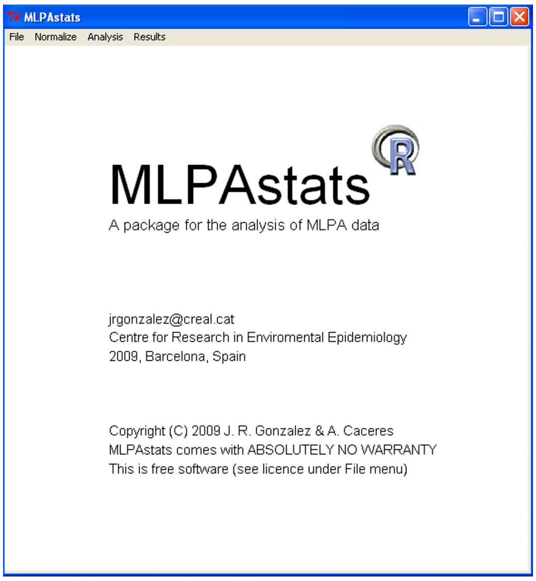

Figure 1: MLPAstats main GUI window.

2 Loading Demo

A sample data can be found in the File menu under the “load demo” option (figure 2). This is data from a breast cancer study (P002 BRCA1) provided by NGRL-Manchester. It consists on a collection of 34 probes for 10 case and 5 control samples. Nine of the probes are used as reference probes for the normalization step.

Loading the data will create an Ms.R file, where the results of your session will be stored. Beforehand, you can change the current directory with “change dir” to the path in which you would like to save that file.

2.1 Exploring the Ms object

This section illustrates how data is loaded onto R. It is not required for the analysis but it will give you an understanding of the data structure. We explore it by using the R command line.

BRCA data is loaded into an Ms variable that contains all the experimental information needed to begin analysis. Type in the command line

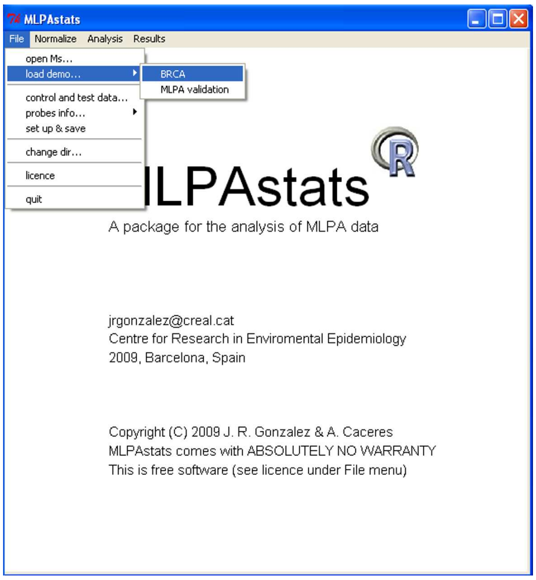

Figure 2: Selection of BRCA demo.

```
> getInfo()

Status of Ms object
-------------------
  Data loaded ...
```

which gives you the status of the Ms variable. When data is loaded Ms is initialized with:

1. **control and test data:** In our example, we have data for 34 probes 5 of wich are control probes

```
> getProbes()

 [1] "C5q31"        "C6p21"        "BRCA1Ex1A"    "BRCA1Ex1B"    "BRCA1Ex2"
 [6] "BRCA1Ex3"     "BRCA1Ex5"     "C15q21"       "BRCA1Ex6"     "BRCA1Ex7"
[11] "BRCA1Ex8"     "BRCA1Ex9"     "BRCA1Ex10"    "C2q14"        "BRCA1Ex11-1"
[16] "BRCA1Ex11-2" "BRCA1Ex12"    "BRCA1Ex13"    "BRCA1Ex14"    "C12p12"
[21] "BRCA1Ex15"    "BRCA1Ex16"    "BRCA1Ex17"    "BRCA1Ex18"    "BRCA1Ex19"
[26] "C4q26"        "BRCA1Ex20"    "BRCA1Ex21"    "BRCA1Ex22"    "BRCA1Ex23"
[31] "BRCA1Ex24"    "C11p13"       "C12p13"       "C3p21"
```

```
> getProbesControl()

[1] "C5q31"  "C6p21"  "C15q21" "C2q14"  "C12p12" "C4q26"  "C11p13" "C12p13"
[9] "C3p21"
```

A closer look to one of the probes shows the values for each subject across all control and test subjects

```
> getPeaks("C5q31", "controls")

  C5q31
1  2204
2  1803
3  2206
4  2423
5  2191
```

```
> getPeaks("C5q31", "tests")
```

```
   C5q31
1   2191
2   1593
3   2763
4   2551
5   1967
6   2324
7   1967
8   1803
9   1803
10  1803
```

2. **probe size:** Each probe requires its size measure for the normalization step, for BRCA data:

```
> getSize()
```

```
 [1] 127 136 148 157 166 175 184 196 208 217 226 235 244 256 268 277 286 295 304
[20] 316 328 337 346 355 364 376 388 397 406 415 424 436 445 454
```

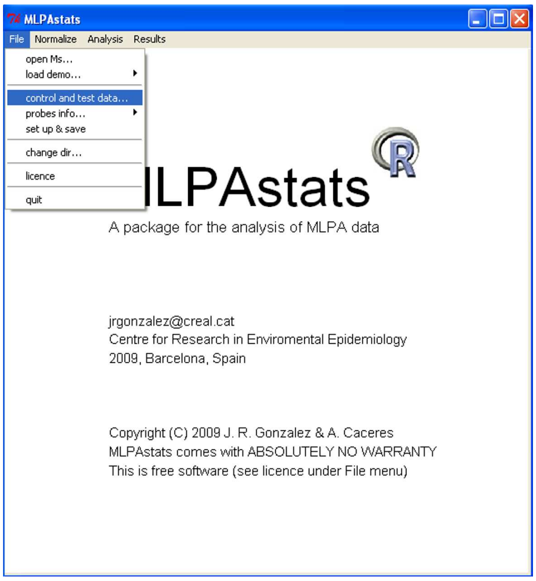

Figure 3: Option fields for making your own Ms.

2.2 Making you own Ms

The steps in this secition have been all automatically set up when you loaded the BRCA demo. If you are following a quick demo you can proceed to the next section, but should come back when trying your own data. For initiallizing the Ms file with your data, you can enter all the required information from text files; we have inlcuded a copy of thse files with the distribution of the package, you can find the location of these files typing

```
> path <- system.file("data", "BRCAcases.txt", package = "MLPAstats")
> path
```

```
[1] "/tmp/RtmpcFxt56/Rinst5f1248a4/MLPAstats/data/BRCAcases.txt"
```

In figure(3), the option “control and test data” directs you to select the data files for the control and case samples. Follow the previous path to identify the required files BRCAcontrols.txt and BRCAcases.txt.

Note that these are text files with data in the format

```
Sample Replica RNAseP.1 HIRA UBE3A.9EX ...
NA.HMP05.a2 A 13220 9108 10644 ...
NA.HMP05.a2 B 13028 9891 10673 ...
...
```

where the first row is the sample, replicates and probe names. Data is entered separated by single spaces. Make sure your own files follow this convention; in particualr, have in mind reserving the first two columns for sample names and replicates, even if you do not have any.

Now you need to specify which of these probes shoul be takes as reference. They can be provided interactively or with a text file. The interactive option is under “select” on the “control probes” submenu; figure (4). The main window, figure (5), displays the probe names to be selected as reference.

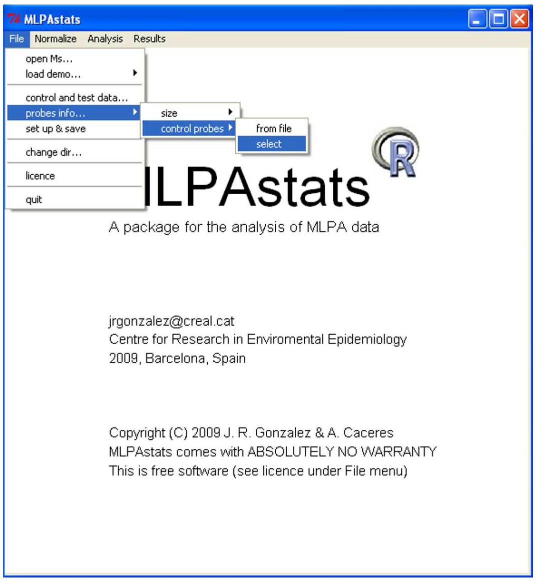

Figure 4: option to select reference probes.

For BRCA data, click on the names that correspond to the reference probes (those starting with C) and then double-click on the OK text.

If the reference probes are entered from a text file, a list of probes numbers corresponding to the controls should be written on a row separated by single spaces. A sample of such file is referecneProbes.txt, that can be selected using the “from file” option.

You can now chose the size of the probes from the “probes info” submenu. The size for the probes can be either typed in or selected from a file menu. The data in the file should consist of a row with the probe numbers separated by single spaces in the same order given in the sample data files. For the data in the BRCA example you can select “probes info”, “size” and “from file” to browse for the file BRCAsize.txt.

After entering all your data, remember to choose “set up & save” under the file menu to save your data into an Ms.R file.

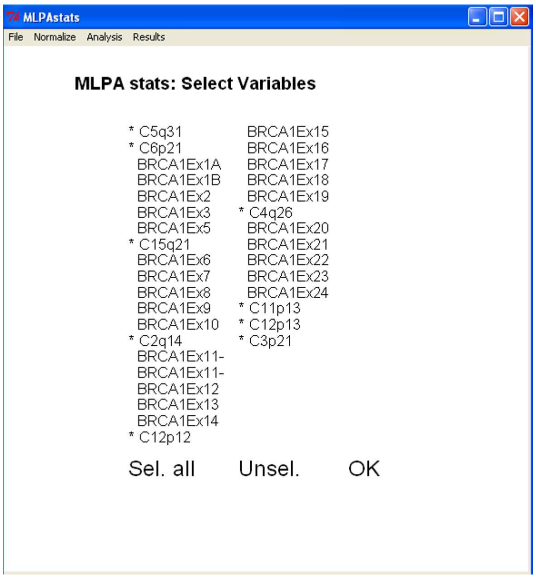

Figure 5: Probe selection for BCRA data.

3 Normalization

Experimental conditions and differences across probes (due to size and nature) can introduce systematic intensity variations that must be accounted for. We have implemented four different approaches to normalize the intensities of the probes. These can be accessed under the Normalize menu, once the Ms object has been initially set up.

The normalization procedures with and without replicas include:

1. *sum peaks control*: In this method normalization is achieved by dividing the peak intensities by the total sum of only the intensity of the reference probes.
2. *sum peaks all*: Here the peak intensities are divided by the total sum of all peaks intensities.
3. *slope correction*: In this normalization option the probe intensity is model as a linear function of the probe size. Extracting this dependence, a normalize set of intensities is obtained.
4. *nonlinear*: This procedure incorporates variability across individuals, requiring data replicates. It is based on a mix model where probe intensities are modelled as function of the probe size, with parameters that account for maximum and asymptotic values of peak intensities, together with the decay rate of intensity with probe size. Each of these parameters is considered as a sum of population-averaged fixed-effects and random-effects that account for individual deviations from these averages. Each probe intensity is finally normalized by dividing its value by its model estimate.

Since the BRCA data has no replicates, you can only normalize it by slope correction or sum of peaks options. Chose slope correction and check the results of the normalization by going to the Results menu and selecting "plot", "normalization" and "mean controls". The main window will display a figure with the mean normalization of the intensities for the

control sample, see figure (6); you can also check the normalization for each individual case sample. In Windows environment the figure is saved on the clipboard so you can paste it directly into any image processing software.

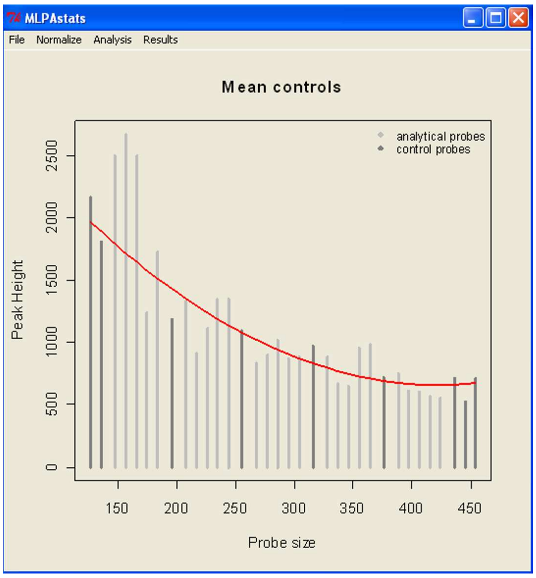

Figure 6: Normalize reults for BRCA data.

3.1 Updating Ms

When you normalize your data the Ms object is updated with the normalization you have selected. The normalization results are stored in the Ms object;

```
> getInfo()

Status of Ms object
-------------------
  Data loaded ...
  Data normalized ...
```

An alternative way to access the plot via the R command line is to type that will give you additional control on the plotting parameters.

Given that the Ms object is automatically saved you can recover your session at any stage by opening a previous Ms object from the File menu.

4 Analysis of Dosage Ratios

Once the data is normalized the inference of the dosage ratios between case and control samples can be done with a variety of methods:

1. *Threshold*: A simple approach to define probes with gains or loses in copy number. Here the ratio between the control and case samples is calculated for each probe and assessed against a loss or gain thresholds (0.7, 1.33).

2. *REX-MLPA*: The Regression-Enhanced MLPA computes the regression between cases and controls with $(\alpha - 1)$ confidence intervals. Test probes with altered copy number are identified as outliers of the confidence region. Starting with the regression of only reference probes the method iteratively includes the probes of the case samples that are within the confidence intervals to re-estimate the regression.

3. *mixel-model*: It is a method for sample replicates that accommodates the error in the control samples and the small number of probes typically involved. A probe has a different copy number for a case sample if their differences with a control samples are greater than the typical difference between controls.

The threshold and the REX-MLPA method can be used for the BRCA data, since they do not require sample replicates. The option “analyze” under the “Analysis” menu opens a window for which you can choose the method of analysis and their relevant parameters.

The results of the analysis can be ploted in MLPA main window. Results for the *Threshold* analysis are shown in figure (7). Plotting REX-MLPA analysis produces a scattered plot of case against control samples for each probe, as shown in figure (8).

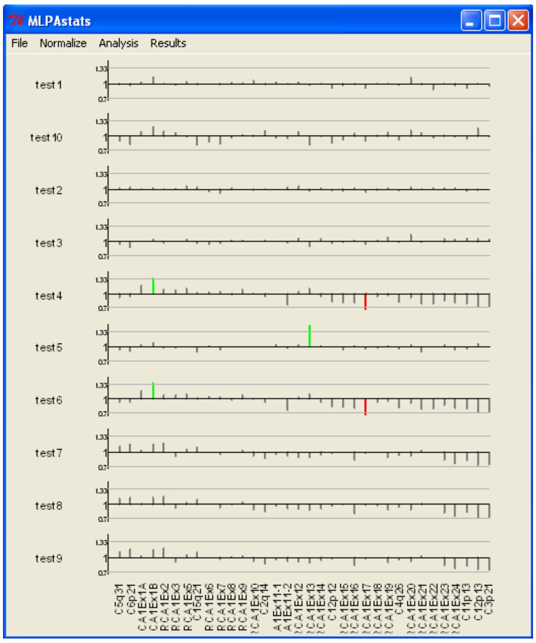

Figure 7: Analysis Results using *Threshold*, green bars indicate gains while red represent copy number losses.

4.1 Results on Ms

The results of your analysis are stored in the Ms object that you can access on a new session. You can check previous results stored in the Ms object which has the following fields:

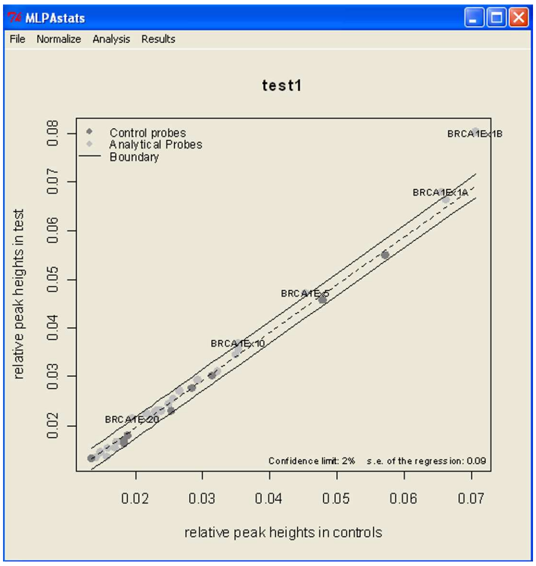

Figure 8: Analysis Results using *REX-MLPA*.

```
> getInfo()
```

```
Status of Ms object
-------------------
  Data loaded ...
  Data normalized ...
  Data analized ...
```

You can recover the analysis configuration typing

```
> getConfig()
```

```
$method
[1] "REX-MLPA"

$bands
[1] "parametric"

$alpha
[1] 0.05

$norm
[1] "slope.correction ; rep= FALSE"
```

And, the final CNV status of each probe for each test sample for such configuration is obtained from the command

```
> getResults()
```

```
MLPA analysis using REX-MLPA (-1: relative loss, 0:normal, 1:relative gain)

       C5q31 C6p21 BRCA1Ex1A BRCA1Ex1B BRCA1Ex2 BRCA1Ex3 BRCA1Ex5 C15q21
case01     0     0         0         1        0        0        0      0
case02     0     0         0         0        0        0        0      0
case03     0     0         0         1        0        0        0      0
case04     0     0         1         1        0        0        0      0
```

```
case05     0     0         1         1        0        0        0      0
case06     0     0         1         1        0        0        0      0
case07     0     0         0         0        0        0        0      0
case08     0     0         0         0        0        0        0      0
case09     0     0         0         0        0        0        0      0
case10     0     0         1         1        1        0        0      0
       BRCA1Ex6 BRCA1Ex7 BRCA1Ex8 BRCA1Ex9 BRCA1Ex10 C2q14 BRCA1Ex11-1
case01        0        0        0        0         0     0           0
case02        0        0        0        0         0     0           0
case03        0        0        0        0         0     0           0
case04        0        0        0        0         0     0           0
case05        0        0        0        0         0     0           0
case06        0        0        0        0         0     0           0
case07        0        0        0        0         0     0           0
case08        0        0        0        0         0     0           0
case09        0        0        0        0         0     0           0
case10        0        0        0        0         0     0           0
       BRCA1Ex11-2 BRCA1Ex12 BRCA1Ex13 BRCA1Ex14 C12p12 BRCA1Ex15 BRCA1Ex16
case01           0         0         0         0      0         0         0
case02           0         0         0         0      0         0         0
case03           0         0         0         0      0         0         0
case04          -1         0         0         0      0         0         0
case05           0         0         1         0      0         0         0
case06          -1         0         0         0      0         0         0
case07           0         0         0         0      0         0         0
case08           0         0         0         0      0         0         0
case09           0         0         0         0      0         0         0
case10           0         0         0         0      0         0         0
       BRCA1Ex17 BRCA1Ex18 BRCA1Ex19 C4q26 BRCA1Ex20 BRCA1Ex21 BRCA1Ex22
case01         0         0         0     0         1         0         0
case02         0         0         0     0         0         0         0
case03         0         0         0     0         0         0         0
case04        -1         0         0     0         0         0         0
case05         0         0         0     0         0         0         0
case06        -1         0         0     0         0         0         0
case07         0         0         0     0         0         0         0
case08         0         0         0     0         0         0         0
case09         0         0         0     0         0         0         0
case10         0         0         0     0         0         0         0
       BRCA1Ex23 BRCA1Ex24 C11p13 C12p13 C3p21
case01         0         0      0      0     0
case02         0         0      0      0     0
case03         0         0      0      0     0
case04         0         0      0      0     0
case05         0         0      0      0     0
case06         0         0      0      0     0
case07         0         0      0      0     0
case08         0         0      0      0     0
case09         0         0      0      0     0
case10         0         0      0      0     0
```

You can again access the plot on your R session

```
> plot(getResults())
```

5 MLPAstats from the command line

If you are comfortable with R the previous analysis can be completed in few steps. First, load the BRCA data with

```
> data(BRCA)
```

and set it up as an object of class setupMLPA

```
> mlpa.dat <- setupMLPA(BRCAcontrols, BRCAcases, size, reference.probes)
```

Second, normalize with the “sum.peaks.controll” option

```
> norm.dat <- mlpaNorm(Ms$mlpa$mlpa.dat, method = "sum.peaks.controls")
```

and check the result plotting:

```
> plot(norm.dat)
```

Finally run and plot the REX-MLPA analysis with the instructions

```
> ans <- mlpa(norm.dat, "REX-MLPA")
> plot(ans)
```

You can check the final copy number assignment for each probe and case sample

```
> ans
```

```
MLPA analysis using REX-MLPA (-1: relative loss, 0:normal, 1:relative gain)

       C5q31 C6p21 BRCA1Ex1A BRCA1Ex1B BRCA1Ex2 BRCA1Ex3 BRCA1Ex5 C15q21
case01     0     0         1         1        0        0        1      0
case02     0     0         0         0        0        0        1      0
case03     0    -1         0         0        0        0        0      0
case04     0     0         1         1        1        0        1      0
case05     0    -1         1         1        0        0        0      0
case06     0     0         1         1        1        0        1      0
case07     0     0         0         0        0        0        0      0
case08     0     0         0         0        0        0        0      0
case09     0     0         0         0        0        0        0      0
case10     0     0         1         1        1        0        0      0
       BRCA1Ex6 BRCA1Ex7 BRCA1Ex8 BRCA1Ex9 BRCA1Ex10 C2q14 BRCA1Ex11-1
case01        0        0        0        0         1     0           0
case02        0       -1        0        0         0     0           0
case03        0        0        0        0         0     0           0
case04        0        0        0        0         0     0           0
case05        0        0        0        0         0     0           0
case06        0        0        0        0         0     0           0
case07        0        0        0        0         0     0           0
case08        0        0        0        0         0     0           0
case09        0        0        0        0         0     0           0
case10        0        0        0        0         0     0           0
       BRCA1Ex11-2 BRCA1Ex12 BRCA1Ex13 BRCA1Ex14 C12p12 BRCA1Ex15 BRCA1Ex16
case01           0         0         0         0      0         0         0
case02           0         0         0         0      0         0         0
case03           0         0         0         0      0         0         0
case04           0         0         0         0      0         0         0
case05           0         0         1         0      0         0         0
case06           0         0         0         0      0         0         0
case07           0         0         0         0      0         0         0
case08           0         0         0         0      0         0         0
```

case09	0	0	0	0	0	0	0
case10	0	0	0	0	0	0	0

	BRCA1Ex17	BRCA1Ex18	BRCA1Ex19	C4q26	BRCA1Ex20	BRCA1Ex21	BRCA1Ex22
case01	0	0	0	0	1	0	0
case02	0	0	0	0	0	0	0
case03	0	0	0	0	0	0	0
case04	0	0	0	0	0	0	0
case05	0	0	0	0	0	0	0
case06	0	0	0	0	0	0	0
case07	0	0	0	0	0	0	0
case08	0	0	0	0	0	0	0
case09	0	0	0	0	0	0	0
case10	0	0	0	0	0	0	0

	BRCA1Ex23	BRCA1Ex24	C11p13	C12p13	C3p21
case01	0	0	0	0	0
case02	0	0	0	0	0
case03	0	0	0	0	0
case04	0	0	0	0	0
case05	0	0	0	0	0
case06	0	0	0	0	0
case07	0	0	0	0	0
case08	0	0	0	0	0
case09	0	0	0	0	0
case10	0	0	0	0	0

MLPAstats is a software package design for easy interaction with the user. Using the R command line, it is possible to get results quickly and reliably. In addition, the GUI offers an easy way to quickly try different analysis scenarios and recover your previous sessions.

6 Acknowledgments

This work has been partly supported by the Spanish Ministry for Science and Innovation (MTM2008-02457).

References

[1] Gonzalez, J.R., Carrasco, J.L., Armengol, L., Villatoro, S., Jover, L., Yasui, Y., Estivill, X. (2008) Probe-specific mixed-model approach to detect copy number differences using multiplex ligation-dependent probe amplification. *BMC Bioinformatics*, **9**, 261.

[2] Mavrogiannis, L.A., Cockburn, D.J. (2004) GeneMaker Software for Multiplex Ligation-Dependent Probe Amplification (REX-MLPA). *Tech rep.*, Yorkshire Regional DNA laboratory [http://leedsdna.info/downloads.htm].
